# Detailed Contact Data and the Dissemination of *Staphylococcus aureus* in Hospitals

**DOI:** 10.1371/journal.pcbi.1004170

**Published:** 2015-03-19

**Authors:** Thomas Obadia, Romain Silhol, Lulla Opatowski, Laura Temime, Judith Legrand, Anne C. M. Thiébaut, Jean-Louis Herrmann, Éric Fleury, Didier Guillemot, Pierre-Yves Boëlle, on behalf of the I-Bird Study Group

**Affiliations:** 1 Sorbonne Universités, UPMC Univ Paris 06, UMR_S 1136, Institut Pierre Louis d’Epidémiologie et de Santé Publique, F-75013, Paris, France; 2 INSERM, UMR_S 1136, Institut Pierre Louis d’Epidémiologie et de Santé Publique, F-75013, Paris, France; 3 Department of Infectious Disease Epidemiology, Imperial College London, London, United Kingdom; 4 Inserm UMR 1181 “Biostatistics, Biomathematics, Pharmacoepidemiology and Infectious Diseases” (B2PHI), F-75015, Paris, France; 5 Institut Pasteur, UMR 1181, B2PHI, F-75015, Paris, France; 6 Univ. Versailles St Quentin, UMR 1181, B2PHI, F-78180 Montigny-le-Bretonneux; 7 Laboratoire MESuRS, Conservatoire National des Arts et Métiers, 75003, Paris, France; 8 Univ Paris-Sud, UMR 0320/UMR8120 Génétique Quantitative et Evolution—Le Moulon, F-91190, Gif-sur-Yvette, France; 9 INSERM U1173, UFR Simone Veil, Versailles-Saint-Quentin University, 78180, Saint-Quentin en Yvelines, France; 10 AP-HP, Hôpital Raymond Poincaré, Service de Microbiologie, F-92380, Garches, France; 11 ENS de Lyon, Université de Lyon, Laboratoire de l’Informatique du Parallélisme (UMR CNRS 5668—ENS de Lyon—UCB Lyon 1), IXXI Rhône Alpes Complex Systems Institute, Lyon, France; 12 Inria—Institut National de Recherche en Informatique et en Automatique, Montbonnet, France; 13 AP-HP, Raymond Poincare Hospital, F-92380 Garches, France; 14 AP-HP, Hôpital Saint-Antoine, Département de Santé Publique, F-75571, Paris, France; Pennsylvania State University, United States of America

## Abstract

Close proximity interactions (CPIs) measured by wireless electronic devices are increasingly used in epidemiological models. However, no evidence supports that electronically collected CPIs inform on the contacts leading to transmission. Here, we analyzed *Staphylococcus aureus* carriage and CPIs recorded simultaneously in a long-term care facility for 4 months in 329 patients and 261 healthcare workers to test this hypothesis. In the broad diversity of isolated *S*. *aureus* strains, 173 transmission events were observed between participants. The joint analysis of carriage and CPIs showed that CPI paths linking incident cases to other individuals carrying the same strain (i.e. possible infectors) had fewer intermediaries than predicted by chance (P < 0.001), a feature that simulations showed to be the signature of transmission along CPIs. Additional analyses revealed a higher dissemination risk between patients via healthcare workers than via other patients. In conclusion, *S*. *aureus* transmission was consistent with contacts defined by electronically collected CPIs, illustrating their potential as a tool to control hospital-acquired infections and help direct surveillance.

## Author Summary

Recent advances in communication technologies allow monitoring high-resolution contact networks. Close proximity interactions (CPIs) measured by wireless sensors are increasingly used to inform contact networks for the dissemination of pathogens in computational models, although empirical justification is lacking. Here, we conducted a longitudinal prospective study for four months in a hospital, including both patients and healthcare workers (HCWs). High-resolution CPIs were recorded continuously, and participants undertook weekly nasal swabs to detect *S*. *aureus* carriage. We set out to test whether the contact network measured by CPIs supported observed transmission episodes. A simulation study was first conducted to choose a test statistic for the association of CPI paths with transmission, showing that CPI path length from transmitter to incident case was the most powerful. Then, we selected patients presenting incident *S*. *aureus* colonization in the data. We showed that CPI paths existed to carriers of the same strain, with path lengths significantly shorter than between random pairs of participants, in agreement with the transmission hypothesis. In-hospital contact networks measured by CPIs inform on opportunities for pathogen transmission. These could be used in surveillance systems to help prevent the spread of nosocomial pathogens.

## Introduction

Chains of transmission in communicable diseases are often identified by *ad hoc* strategies, combining retrospective information on locations attended and pathogen genetics to identify time-consistent transmission paths.[1] In contrast with such undertakings, “digital epidemiology” propose to use new technologies to prospectively measure contacts and understand transmission[2,3]. Close-proximity interactions (CPIs) between persons recorded by wireless sensors[4] in real-life settings like schools or hospitals[5,6] have been used as indicators of contact in this respect. However, there is no evidence yet that such CPIs actually capture contacts explaining transmission.

To test this hypothesis, we designed a study where both *Staphylococcus aureus* carriage and CPIs were measured in a 200-bed long-term care facility with 5 wards. This setting has several advantages for our purpose: *S*. *aureus* is commonly found in healthcare facilities, colonizing patients and healthcare workers (HCWs); *S*. *aureus* carriage in the nares is usually prolonged as the nares are the most consistent area from which it can be isolated,[7] allowing its detection by repeated routine screenings; identical genetic and antibiotic-resistance profiles show that *S*. *aureus* strains spread among patients and HCWs[8,9]; long-term care facilities harbor a stable population, with patients staying for extended periods under the care of dedicated staff. The control of *S*. *aureus* transmission is also relevant for hospital hygiene, because its carriage increases the risk of healthcare-associated infections.[10]

In our study, *S*. *aureus* carriage was identified in patients and, importantly, in HCWs every week by repeated nasal swabbing. During the same period, all participants wore small wireless sensors that recorded their CPIs with each other in real time (every 30 s). To make the best use of this new data and account for the difference in temporal granularity, we first assessed the ability of several statistics to test the correlation of CPI records and *S*. *aureus* carriage. These characteristics are first presented based on the analysis of simulations where a pathogen spread according to the CPI network edges, then applied to the original data.

## Materials and Methods

### Ethics statement

Authorizations were obtained in accordance with French regulations regarding medical research and information processing. All French IRB-equivalent agencies accorded the i-Bird program official approval (CPP 08061; Afssaps 2008-A01284-51; CCTIRS 08.533; CNIL AT/YPA/SV/SN/GDP/AR091118 N°909036). Signed consent by patients and staff was not required according to the French Ethics Committee to which the project was submitted.

### Data collection

The I-Bird (individual-based investigation of resistance dissemination) study was conducted in a 200-bed long-term and rehabilitation hospital in northern France. The hospital is organized in 5 wards corresponding to medical specialties (geriatrics, neurology, nutrition, orthopedics, post-operative care). During the study period, 329 distinct patients stayed in the facility. Hospital staff (HCWs and other administrative personnel) numbered 261. In the text, all hospital staff is referred to as healthcare workers (HCWs). More details about the I-Bird investigation are provided in S1 Text. When relevant, patients, nurses, nurses’ aides and physicians were analyzed according to the ward in which they stayed or worked; night-shift staff, reeducation therapists and administrative personnel were excluded from these analyses as they were not assigned to a particular ward.

During the study period, all individuals (patients and HCWs) wore a small wireless sensor that recorded, every 30 s, the identity of other sensors that were in close proximity (typically < 1.5m, front-facing). The deployment of such sensors did not rely on any stationary infrastructure to record CPIs, as each sensor directly stored timestamped CPIs on its on-board flash memory. Further details on CPI collection and network reconstruction, along with descriptive characteristics of contact patterns, are available in S2 Text. In the following, analyses are conducted on a dynamic CPI network aggregated at a daily scale by pair of individuals (*ie* network edges). We defined an individual’s *k*-hop neighborhood as all other individuals in the network who were found within *k* steps from him. For example, the 1-hop neighborhood of an individual contained all his direct neighbors, while his 2-hop neighborhood contained both his direct neighbors and these neighbors’ neighbors.

### Defining incident *S*. *aureus* colonization episodes and CPI-supported transmission paths

All participants underwent weekly nasal swabs to monitor *S*. *aureus* carriage. Upon detection of *S*. *aureus* colonization, the isolated strains were *spa*-typed[11,12] and their resistance profiles to 20 antibiotics were determined (see also S1 Text for detailed protocol). The screening procedure had an expected sensitivity of 61.5% and specificity of 98.8%,[13] although higher sensitivity value has been reported (∼80%).[14] The anterior nares were preferred to other body areas because they harbor the most stable *S*. *aureus* colonization and also reflect on overall body carriage.[7,15] Furthermore, eradication of nasal carriage is also associated with eradication of skin carriage.[16,17] *S*. *aureus* strains were considered identical when they had the same *spa* type and antibiotic-resistance profile, in accordance with studies comparing *spa* typing to other molecular techniques.[11,12,18,19] Transmission events were identified by the isolation of a new *S*. *aureus* strain from a patient’s swabs, defining “incident colonization episodes”. Because HCWs may be transiently colonized,[8,20,21] which would mostly be missed with weekly swabs, we only considered incidence in patients. To account for imperfect *S*. *aureus* detection in case of multiple carriage, we also required that the new *S*. *aureus* strain had not been detected in the patient’s previous 2 swabs had he been colonized with another strain in the preceding week.

Each incident colonization episode was investigated to identify time-consistent CPI paths linking the incident case to a previous carrier of the same strain. Recent CPI paths were favored over others by applying the following algorithm:

- All individuals carrying the same strain in the three preceding weeks were defined as “candidate transmitters”, regardless of their CPI connections.
- CPI paths to all candidate transmitters were looked for. In case of existence, the candidate transmitter became a “CPI-supported candidate transmitter”. In this case, the CPI path length (in hops) was computed.
- We sorted all CPI-supported candidate transmitters according to distance in time, then distance in hops. The first CPI-supported candidate transmitter in this list was the “CPI supported transmitter”. In other words, it was the least remote in number of hops among all candidate transmitters arising the least remote in time. In case of ex aequo, one of the candidate transmitters was chosen at random if required.

We investigated 3 weeks before incidence as it allowed finding a CPI-supported transmitter for all incident episodes but 4, which were not CPI-supported by exploring further back in time.

### Statistical analysis

**Testing for CPI supported transmission: test statistics & simulations.** We identified three observable quantities that would provide evidence for the correlation between CPIs and observed transmission: S1—The proportion of incident colonization episodes with least one CPI-supported transmitter; S2—The proportion of CPI-supported transmitters in direct CPI with an incident case; and S3—the length of the shortest CPI-supported transmission path (a good proxy to the actual transmission path[22]). Each of these characteristics can be used to build a test, where, classically, observations would be compared to the expected values under the null hypothesis of independence between CPIs and transmission. These expected values can be computed by a Monte Carlo approach: carriage information was first randomly permuted between participants. To keep autocorrelation between successive swabs in the same individuals, we permuted carriage information over the 3 preceding weeks simultaneously. As *S*. *aureus* prevalence was similar between patients and HCWs (Table 1), we did not take occupation into account for permutations. For each incident colonization episode, 100 replicates of permuted carriage statuses were generated to simulate the distribution of statistics of interest for investigated strategies.

**Table 1:** Characteristics of participants, close proximity interactions and *S*. *aureus* carriage.

	**Patients**	**HCW**
**Total number**	329	261
**Sex (% male)**	43.5%	42.5%
**Age (median [range])**	58.5 [24.5–102.8]	41.3 [18.7–61.3]
**CPI number per d**	12 (± 6.2)	15 (± 7.2)
**With patients**	6 (± 4.5)	9 (± 5.7)
**With HCWs**	6 (± 2.5)	6 (± 3.1)
**CPI cumulative duration (h/d)**	12.2 (± 11.3)	3.7 (± 2.4)
**With patients**	11.1 (± 11)	1.7 (± 1.3)
**With HCWs**	1.1 (± 1.6)	2 (± 1.7)
**1-hop neighborhood**		
**Size**	76 (± 48)	NA
**Time to 50%**	5 days (± 8)	NA
**Time to 75%**	14 days (± 14)	NA
***S*. *aureus* carriage prevalence**	38% [35.3–40.7%]	36.3% [32.3–40.4%]
**1-month cumulative *S*.*aureus* incidence**	33% [25–41%]	NA

Values are mean ± SD or percent [95% C.I.], unless stated otherwise. NA, not applicable.

For S1 and S2, we compared the observed percentages of CPI supported paths to that expected after random permutations of carriage (e.g. in S1, the observed percentage of CPI-supported episodes was compared to the average proportion of CPI-supported episodes among all permutations). For S3, the shortest CPI path length was averaged across all replicates for each incident colonization episodes, thus providing the expected distribution under the null. The observed CPI path length distribution was then compared to that expected under the null using the Wilcoxon signed rank paired test.

To first study the characteristics of the three approaches and choose the most powerful, we used a simulation study based on a Susceptible—Colonized—Susceptible transmission model on the CPI network. Stochastic simulations were performed to mimic observations of incident colonization episodes in our study. First, the dynamic CPI network between all participants in a random 3-week long period was selected. All individuals in this network were assumed initially noncolonized (i.e. susceptible), except one randomly chosen to be the initial carrier in the first week. For each day *d* in the following 3 weeks, a noncolonized individual (say *i*) could become colonized with probability

$$P_{i}\left(d\right)=1-\left[{\left(1-P_{PA}\right)}^{n_{PA}\left(i,d-1\right)}\;*\;{\left(1-P_{HCW}\right)}^{n_{HCW}\left(i,d-1\right)}\right]$$

where *n*_*PA*_(*i*, *d*-1) is the number of carrier-patient neighbors on day d-1 and *n*_*HCW*_(*i*, *d*-1) the number of carrier-HCW neighbors of *i*, *P*_*PA*_ the probability of transmission per contact with a carrier patient and *P*_*HCW*_ with a HCW. Colonized individuals cleared colonization at a constant rate *q*_*PA*_ = 0.1 days^−1^ for patients and a *g*_*HCW*_ = 0.45 days^−1^ for HCWs in agreement with other studies.[23,24] The observation model closely imitated those of our investigation: from the wholly simulated transmission chain, we only selected data determining carriage once a week in each participant. As in practice, the status of participants in the same ward was determined the same day. Finally, an incident colonization episode was selected from new carriers in week 3 of the simulation, as in the original data. Simulations were run to produce observations of a variable number of incident cases. The power of each statistical approach, S1 to S3, was determined as a function of the number of incident episodes.

#### Survival analysis of *S*. *aureus* colonization in patients

The time to colonization in patients was studied by survival analysis. Time was counted from admission to first colonization episode and censored at discharge otherwise. A Cox proportional hazard regression model was used to assess the risk of colonization with respect to network-related covariates, including the numbers of weekly CPIs (*ie* an individual weekly degree) and their cumulative durations, which were included as time-dependent.

#### Risk of transmission through patients or HCWs

The relative risk (RR) of transmission via a HCW *versus* a patient was estimated by the ratio of the percentage of intermediary HCWs in CPI paths leading to transmission to that not leading to transmission. For each candidate transmitter at 2 hops from an incident case, we determined how many 2-hop neighbors were in the CPI network, and whether they were incident cases.

All statistical analyses were conducted with R v3.0.1.

## Results

**Fig 1:**
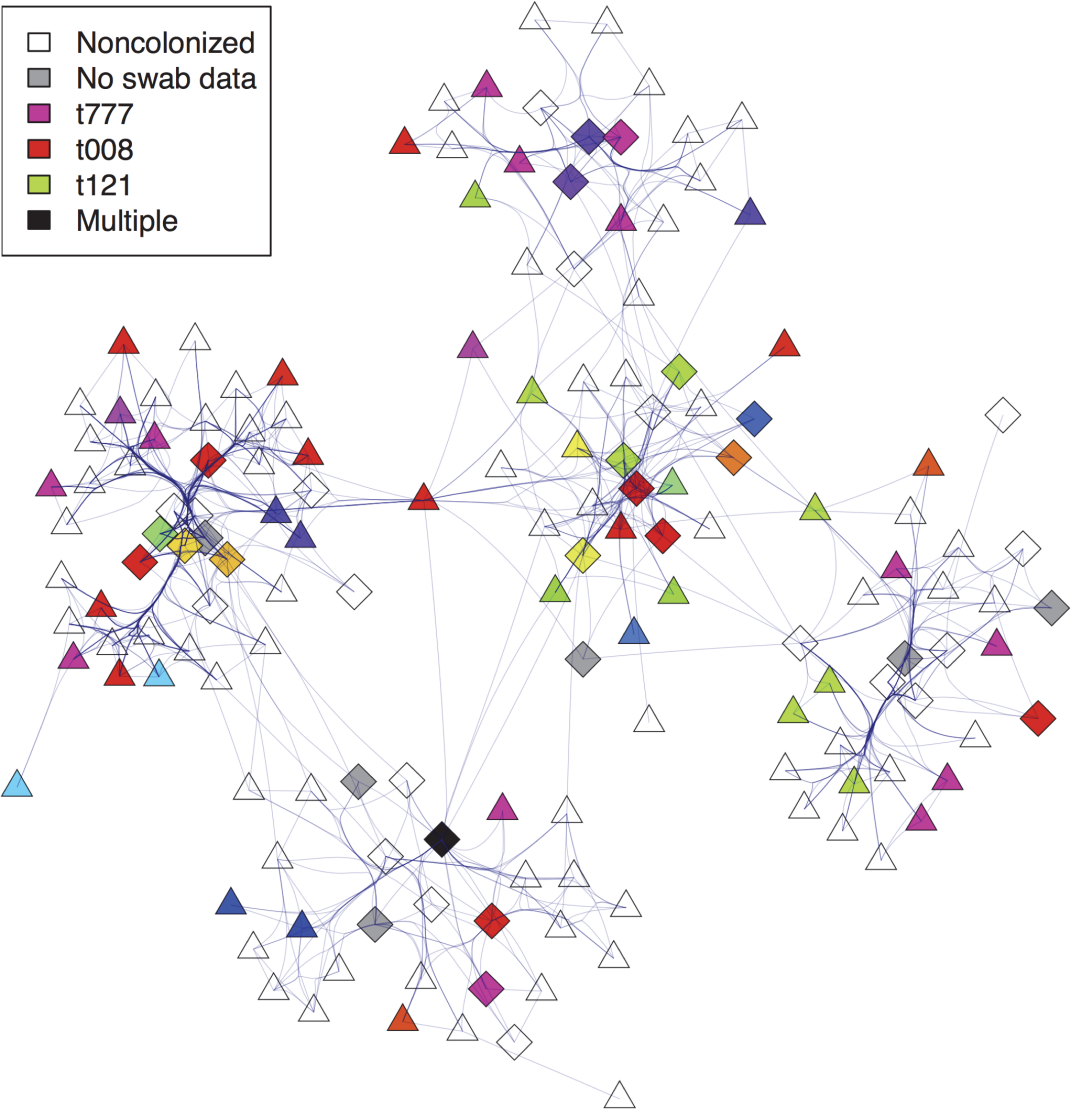
CPI-based network and *S*. *aureus* carriage in the hospital. The network shown corresponds to interactions occurring during 1 d. Patients are shown as triangles and HCWs as diamonds. Color-coding corresponds to the *spa* type of the last known colonization status (i.e. the preceding week). Because of the large *S*. *aureus spa* types diversity, only the 3 most common are reported in the legend. We used Fruchterman-Reingold force-directed algorithm for the layout (individuals in the network are closer together as the density of links among them increases). Force-directed edge bundling was used to accommodate their high density.

**Fig 2:**
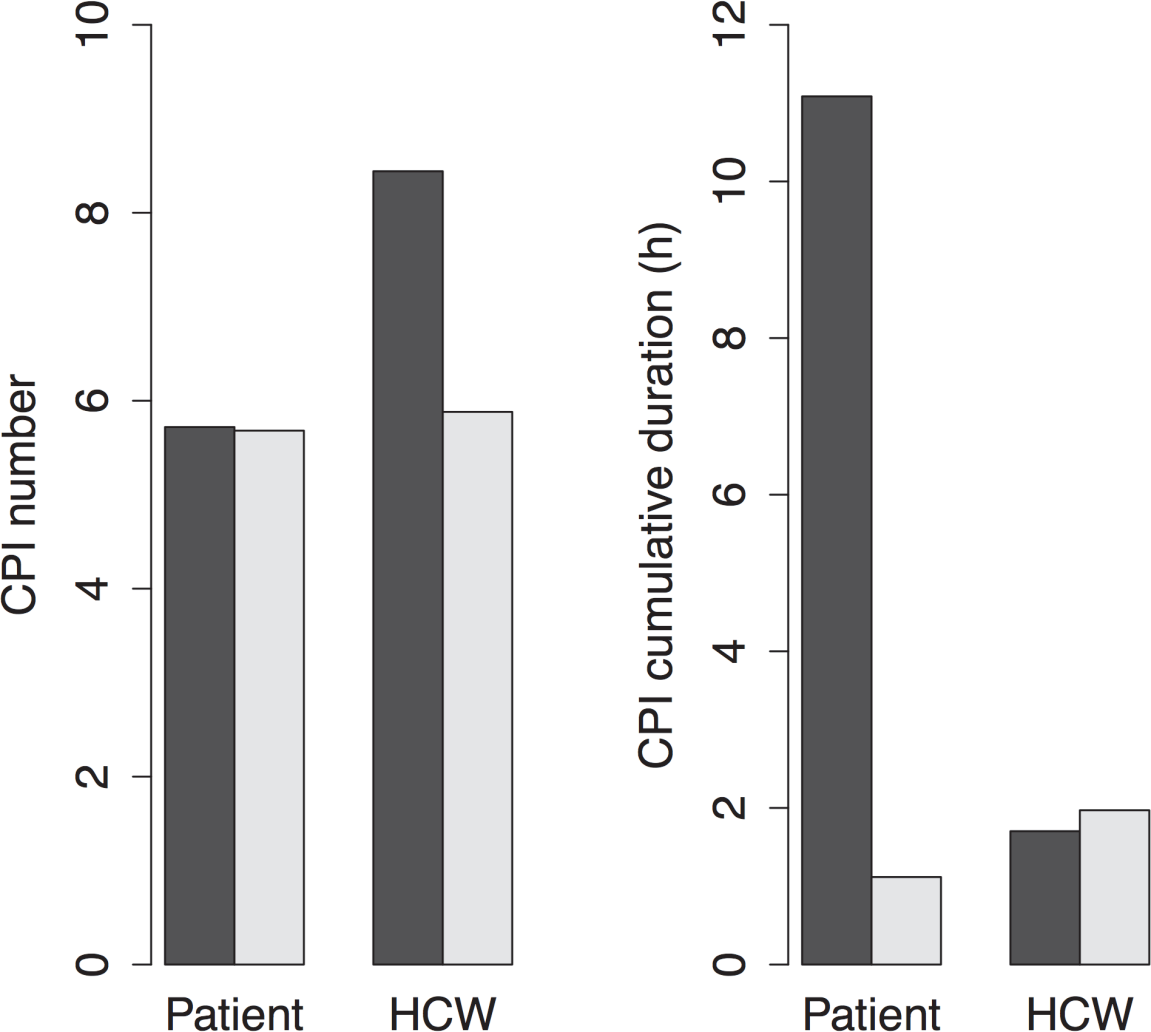
Mean daily numbers and durations of patients’ and HCWs’ CPIs. Bars illustrate CPIs with patients (black) and HCWs (white).

CPIs were recorded among 590 individuals (329 patients and 261 HCWs) during the 4-month period (Table 1), and yielded 85,025 daily CPIs. Each day, a CPI network was defined with study participants as nodes and CPIs as edges (Fig. 1). The collection of daily CPI networks defined the “dynamic CPI network”. While the numbers of CPIs were within the same range for patients and HCWs, the daily-cumulative durations of the CPIs were much longer for patients than HCWs, respectively: 12.2 (± 11.3) h (mean ± SD) vs. 3.7 (± 2.4) h (Fig. 2). Further description of the dynamic CPI network is provided in S2 Text.

During the same time, 4,175 swabs were collected: 37.2% of them (1550 swabs from 363 participants) were positive for *S*. *aureus* carriage. In all, 148 different *spa* types were isolated during the study (Fig. 1, and Fig. 1 in S1 Text for incidence per week and *spa* types). Notably, 114 strains were isolated more than once, each in three participants on average, suggesting that transmission had occurred among these individuals.

### A simulation study of tests for the correlation of CPI-supported paths and carriage

We assessed the power of all proposed strategies with increasing numbers of incident colonization episodes (10, 20, 30, 50, 100 and 153). For each of these amounts, 500 replicates of 100 permutations were performed. Each strategy was performed on every replicate. Table 2 shows the power of the tests to reject the null hypothesis of independence between CPIs and transmission. Strategy S1, based on the existence of CPI supported transmitters, yielded very poor results. Indeed, in almost all situations, a CPI-supported transmitter existed in the original data as well as in the permuted data, so that no difference from random was seen in this characteristic. The percentage of incident cases in direct CPI with CPI-supported transmitters and the shortest CPI-supported path length yielded more useful procedures. The length of the shortest CPI path from transmitter to incident case (S3) was slightly more powerful than the percentage of transmitters in direct CPI with the incident case (S2), although both approaches had large power for rejecting the null for samples of size 153.

**Table 2:** Statistical power computed for the three proposed test statistics.

				Power (%)		
	*n = 10*	*n = 20*	*n = 30*	*n = 50*	*n = 100*	*n = 153*
**Test statistics**						
CPI-supported paths (S1)	0	0	0	0	0	0
CPI-supported transmitter in direct contact (S2)	0.67	0.93	0.98	0.99	1	1
CPI-supported transmitter path length (S3)	0.75	0.96	0.99	1	1	1

Power was determined for an increasing set of incident colonization episodes, with 500 replicates each time.

### Carriage of *S*. *aureus*

The time to first *S*. *aureus* colonization was analyzed for 201 patients who were not colonized at admission: 73 experienced incident colonization. The cumulative incidence of *S*. *aureus* colonization was 33% (95% confidence interval (C.I.) [25–41%]) 1 month after admission, with almost equal incidence of methicillin-resistant *S*. *aureus* (MRSA) (23.2% (95% C.I. [15.1–30.6%])) and methicillin-sensitive *S*. *aureus* (MSSA) (16.5% (95% C.I. [9.3–23.2%])). The risk of colonization did not change with the number of distinct direct neighbors during the preceding week (*ie* weekly degree; hazard ratio (HR) = 1.05 (95% C.I. [0.95–1.21]) for a 5-neighbor increase, *P* = 0.4), using either the raw number of CPIs (*ie* the sum of daily degrees; HR = 1 (95% C.I. [0.95–1.10]) for a 5-CPI increase, *P* = 0.4) or the cumulative duration of CPIs (*ie* the weight of network edges; HR = 1 (95% C.I. [0.99–1.01]) for a 1-h increase, *P* = 0.6). The same conclusions were drawn for MSSA or MRSA colonizations.

Overall, 237 incident-colonization episodes were documented in 111 patients (144 MRSA, 93 MSSA). For each incident episode, we identified “candidate transmitters”, i.e. people who had carried the same strain at any time in the preceding three weeks. Among the 237 incident-colonization episodes, 173 (73%) had 307 candidate transmitters, with no difference between MRSA and MSSA episodes (76% (110/144) vs. 68% (63/93), *P* = 0.16). Episodes without a candidate transmitter did not occur earlier post-admission than others (8.1 *vs*. 8.3 weeks, *P* = 0.8), or preferentially in some wards (*P* = 0.13). Twenty of the 173 episodes with candidate transmitters were discarded as the incident case had a missing CPI record due to sensor failure in the preceding weeks.

### Testing for CPI support

**Fig 3:**
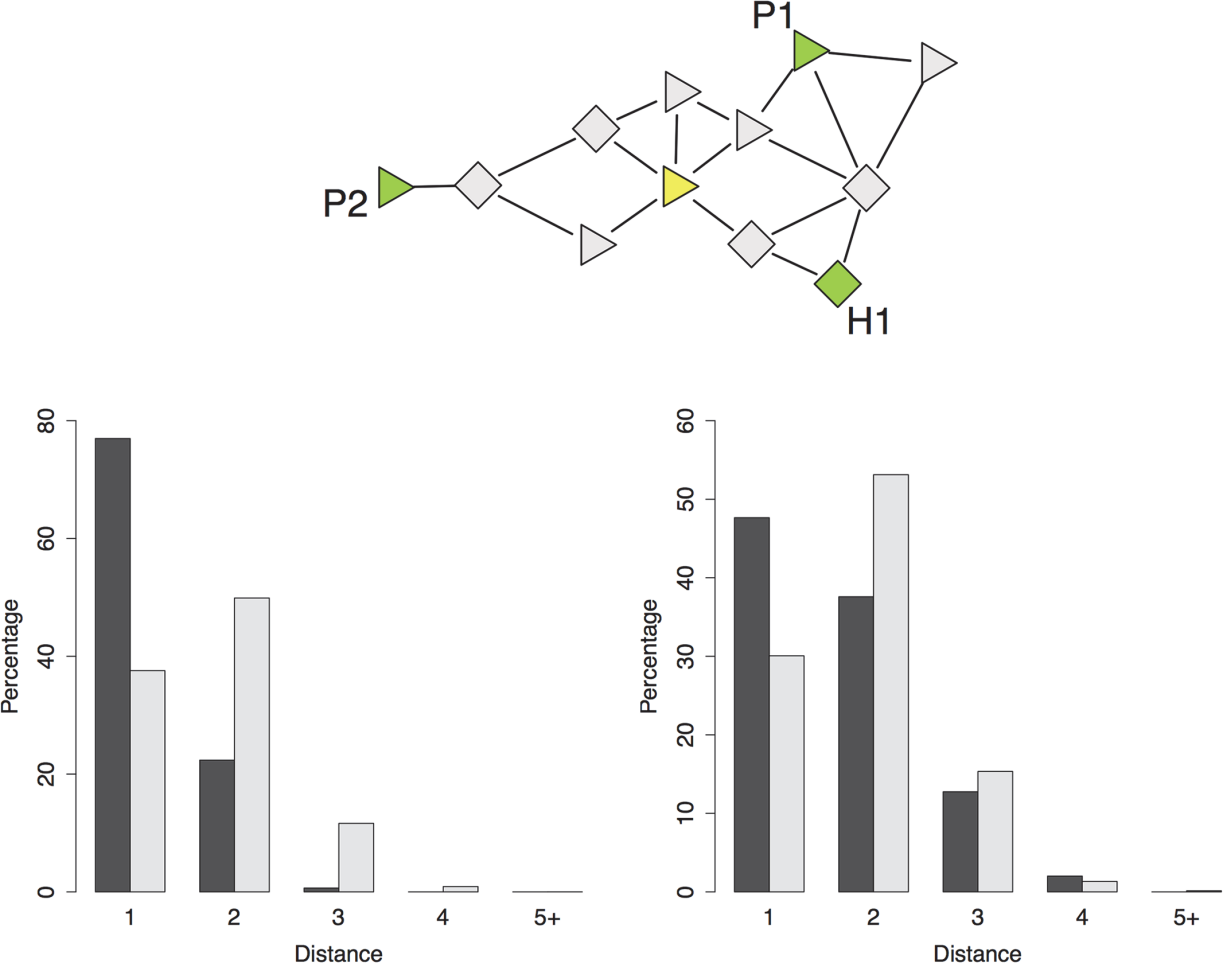
CPI supported transmitters. **(Top)** CPI-supported transmitters were selected among carriers of the same strain (green) as the incident case (yellow) who were the closest in the CPI network. Here, P1 and H1 are two CPI-supported transmitters in the incident case’s 2-hop neighborhood, but not P2 who is further away (3-hop neighborhood). Patients are shown as triangles and HCWs as diamonds. **(Bottom left)** Comparison of the distance distribution between CPI-supported transmitters and incident cases in the data (black) and with random permutations of carriage data (white) in the simulation study. **(Bottom right)** Comparison of the distance between CPI-supported transmitters and incident cases in the original data.

We investigated all 153 incident colonization episodes detected from longitudinal swab data. As expected from the simulations, a CPI path existed between the candidate transmitter and the incident case in almost all instances (97%, Table 3). The characteristics of the shortest CPI paths lengths from candidate transmitter to incident case were in favor of transmission along CPIs, with shorter paths in the original data than after random permutations (Fig. 3, Strategy S3: *P* < 0.001). This was therefore the sign that *S*. *aureus* transmissions detected from the I-Bird swabs were driven by CPIs.

**Table 3:** Incident-colonization episodes, candidate transmitters and CPI support.

**Incident-colonization episode**	***S*. *aureus***	**MRSA**	**MSSA**
**All, n**	237	144	93
**With ≥ 1 candidate transmitter(s), n**	173	110	63
With available CPI network*, n	153	100	53
**CPI-supported†, n**	149 (97%)	99 (99%)	50 (94%)

Incident-colonization episodes were investigated when the incident strain had been isolated from at least one another participant (candidate transmitter). When a CPI path existed between a candidate transmitter and the incident case, the episode was considered as CPI-supported.

* Episodes where CPI were not recorded for the incident case were discarded

† Among those with available CPI network

A direct CPI contact existed between the candidate transmitter and incident case (i.e. a CPI path of length 1) in 48% of the cases *vs*. only 30% expected by chance (Strategy S2: *P* < 0.0001). A CPI path of length 2 was observed in 38% (*vs*. 53%) of the episodes and of length larger than 2 in the rest of the cases.

In most cases (64%), a CPI supported transmitter was found in the preceding week. The remaining were found in the preceding two (23%) or three weeks (13%).

### Increased transmission via HCWs

We next investigated transmission differences according to occupation, focusing on dissemination events for which the path from candidate transmitter to incident case had exactly one (noncolonized) intermediary. For these 2-hop CPI paths, *S*. *aureus* spread was more frequently observed when the intermediary was a HCW than a patient (2.1% vs. 1.8%, *P* = 0.0004). The relative risk (RR) of transmission by a HCW was therefore 1.2 (95% C.I. [1.1–1.3]). This increased risk was more pronounced when the initial carrier was a patient (1.9% vs. 1.5%, *P* < 0.0001; RR = 1.3 (95% C.I. [1.1–1.4])) rather than an HCW (3.2% vs. 3.0%, *P* = 0.48; RR = 1.1 (95% C.I. [0.9–1.2])).

### Sensitivity analyses

All analyses were repeated using a thinned dynamic CPI network obtained by excluding short individual interactions lasting < 5 min prior to daily aggregation. The thinned CPI network was still dense, including 8.1% of all potential interactions. As expected, this decreased density increased the number of intermediaries between 2 persons (mean = 11 ± SD = 6) compared to the full network and decreased the number (mean = 4 ± SD = 1) and duration of daily CPIs. The distribution of within/outside-ward CPIs was almost the same as before, with 75% of CPIs occurring within the ward. Again, no risk factor that could increase the risk of colonization was identified.

The CPI support of transmission was even larger than before, with shorter path lengths in the original network than expected by chance (*P* < 0.0001). In the thinned network, 64% of candidate transmitters were in the incident case’s 2-hop neighborhood (i.e. direct or with one intermediary), compared to 46% with the permuted carriage data.

Keeping all candidate transmitters with a path to the incident case, rather than only the closest ones did not change the conclusions. Excluding repeated incident episodes of the same strain in a given patient, leading to consider 129 transmission episodes only, did not affect the conclusions drawn regarding CPI support. Finally, keeping only episodes that were CPI-supported in the first week before incidence led to the same conclusion (96 CPI-supported episodes, CPI path length significantly shorter (*P* = 0.01)).

To account for imperfect sensitivity and possible false negatives in swabs, we discarded all incident episodes where the carriage had been positive/negative/positive with the same strain at both ends, as those may be false negatives. This led to retain 129 incident colonization episodes with candidate transmitters and CPI records, among which 126 were CPI supported (ie 98%). The shortest CPI path length was significantly shorter than chance predicted (P < 0.0001).

## Discussion

The contribution of the contact network between patients and HCWs in explaining hospital-associated infections is widely accepted[25,26], although it has never been tested empirically. Here, using electronic wireless devices to record close proximity interactions among persons in a hospital, we find evidence that these interactions are indeed informative for *S*. *aureus* transmission. To date, such high-resolution contact networks were used to structure contacts in computerized models and study how characteristics of individuals[27,28] and network topology[2,29] could influence the course of outbreaks. However, these findings relied on the underlying hypothesis that CPI paths actually captured dissemination paths. Our results provide evidence that CPIs recorded by electronic sensors are indeed relevant to explain transmission. This validates using such networks in future epidemiological studies, and should provide a powerful tool to better characterize risk and plan control measures for pathogens transmission in specific settings.

Recording interactions among individuals is increasingly easy using remote sensors[30]. In the study facility, wearing sensors was well accepted by patients and HCWs. Because the recording was limited (typically < 1.5 m), these signals may be a good proxy for real-life contacts between people. Although contact surveys were shown to document contacts hardly overlapping with those recorded by electronic sensors,[31] direct observations by dedicated investigators provided more congruent data.[32] Individual surveys tend to omit contacts of short duration,[31] and therefore electronic sensors might capture more complete data regarding contact patterns. In our study, although most CPIs occurred within the wards (leading to clustered communities, as seen in Fig. 1), the CPI network was rather dense, covering up to 20% of all possible interactions among participants. The shortest path between any two individuals had few intermediaries, a typical “small-world” feature[33]. HCWs spent approximately 20% of their work shifts in direct contact with patients (1h50 out of 8h), in the same range as that reported in an emergency unit (∼30%)[34] and the cumulated CPIs duration in a HCW compared with that reported for another ICU (∼2.2 h)[32]. However, in sharp contrast with an earlier study conducted in a pediatric setting[30], where almost no contacts existed between patients, CPIs between patients herein were frequent and prolonged, as expected in a long-term care and rehabilitation facility where patients can initiate social interaction with others more easily than in acute-care hospital. Finally, ward organization was important in structuring contacts between patients and HCWs. This contact-clustering suggests that some interaction patterns between patients and HCWs could be rather constant from one hospital to the next, e.g., the numbers and durations of interactions, but that the full contact network may depend on type of care and ward organization. Other features of interest were the quick encounter of most of direct contacts during the first week after admission and that an incoming patient’s 3-hop neighborhood quickly encompassed almost all individuals in the hospital. This small-world feature may profoundly affect the potential spread of pathogens, increasing the size and speed of outbreaks[33,35].

*S*. *aureus* carriage was common among patients and HCWs, with a significant percentage of patients already colonized at admission (33.7% for *S*. *aureus*; 17% for MSSA; 18.9% for MRSA). Approximately one-third of noncolonized, newly admitted patients became colonized with *S*. *aureus* within the first month post-admission, as frequently observed in long-term care facilities[36,37]. The cumulative MRSA and MSSA incidence rate were similar after 1 month, as were their mean carriage prevalence, suggesting little, if any, difference in transmission between resistant and non-resistant strains. Patients admitted to long-term care facilities come from other hospitals and prevalence of carriage at admission is large. We analyzed incident carriage episodes only to focus on transmission occurring within the long-term care facility. The numbers and durations of contacts, although pointing towards increased risk for participants with more and longer contacts, were not by themselves strongly associated with *S*. *aureus* colonization: additional information on whether the contacts were carriers is likely to be required in this respect. Finally, because only weekly nasal swabs were conducted, neither hand carriage nor transient colonization episodes (<1 week) were considered, although hand carriage by HCWs has been described[8]. In our analysis, this may lead to imperfect observation of the carriage status, as participants may have cleared colonization between successive swabs.

In our study, *S*. *aureus* carriage was determined by nasal swabs. As previously stated, skin colonization was not detected. Yet, previous studies have shown that while *S*. *aureus* can be isolated from many anatomic location, nasal swabs were consistent with carriage isolated from other area of the body in 82% of the case[38]. In HCWs, where transient colonization is more likely to occur, this might lead to underestimate prevalence and therefore overestimate incidence, as an (unobserved) skin carriage could lead to a longer, more stable, colonization of the nares. For this reason, we chose not to include incident colonization episodes occurring in HCWs. Although the main route of transmission for *S*. *aureus* remains physical contact with carrier individuals, transmission through the environment is also possible, for example in the form of fomites.[26] Our procedure cannot distinguish between routes of transmission leading to short CPI paths, for example, if contact with fomites occurred only when people were at CPI range of a known carrier. Yet, our results suggest that CPIs, as defined in our study setring, correlated with *S*. *aureus* transmission routes and are therefore a good proxy[35] for interactions leading to *S*. *aureus* dissemination.

The choice of a test statistic to test for transmission along CPIs required giving some consideration to the setup of this study. First, the density of contacts between participants was large, as in occupational networks measured in health care structures.[24,30] This leads to percolation,[39] with typical short distance between participants. Therefore, a CPI path between a *S*. *aureus* carrier and an incident case was not specific enough of transmission: it was the rule rather than the exception, explaining why strategy S1 could not evidence association. Second, while CPIs could be recorded continuously, it is obvious that *S*. *aureus* carriage cannot be determined as frequently. Some participants could therefore clear carriage between two successive swabs, hiding their role in transmission. In this case, direct CPI would not be seen in the recorded CPIs. However, a short CPI path may be found to a more distant carrier through non-colonized intermediaries and would still be supportive of transmission. The shortest CPI path between CPI-supported transmitters and incident cases allowed to account for carriage gaps in observed transmission chains and was more informative than mere existence of a connecting path. The power comparison between strategies S1, S2 and S3 showed that this was indeed the case. It also evidenced that the proposed tests actually discriminated between transmission along the CPI paths and random transmission with no relationship to the CPI network.

In contrast to weekly swabbing, wireless sensors recorded interactions permanently and made it unlikely that the network of interactions was imperfectly observed. *S*. *aureus* transmission is thought to occur mainly through physical contacts and these should be present in the CPI recordings. However, the CPI network may capture additional interactions that are unlikely to lead to transmission. To focus on interactions that were the most likely to lead to transmission, for example nursing care, we discarded all CPIs lasting less than 5 min. In this thinned network, we found again evidence that paths defined by CPIs supported transmission. Finally, the increased likelihood of *S*. *aureus* spread through a—seemingly noncolonized—HCW intermediary was in good agreement with their importance in transmission and the occurrence of transient colonization among them.

The hypothesis that CPIs are a good proxy for contacts leading to transmission of *S*. *aureus* is highly plausible *a priori*. Indeed, the main route of transmission for *S*. *aureus* is physical contact; those led to CPI records as sensors recorded all physical proximity (<1.5m). The mechanism of transmission was therefore captured by CPIs. With the evidence we provide on the correlation between CPI paths and transmission events, this strengthens the interest of this proxy measure as a cheap, feasible and informative method for studying *S*. *aureus* transmission.

## Conclusion

The joint analysis of *S*. *aureus* carriage and CPI data collected during 4 months provided evidence that CPIs capture contacts associated with transmission. This supports using CPI information to improve the realism of transmission models. This also suggests that a more systematic in-depth study of CPI networks could provide new directions for controlling *S*. *aureus* transmission in hospitals.

## Supporting Information

S1 Text I-Bird investigation methods. (DOCX)

S2 Text Description of the dynamic CPI network. (DOCX)
