# Supplementary material for: Detailed Contact Data and the Dissemination of Staphylococcus aureus in Hospitals
Source: PLoS Comput Biol. 2015 Mar 19;11(3):e1004170. doi: 10.1371/journal.pcbi.1004170 (PMC4366219; doi:10.1371/journal.pcbi.1004170)

# S1 Text: I-Bird investigation methods

## Design and participation

The I-Bird investigation was a prospective, observational, cohort study conducted in a 200-bed long-term care hospital over a 6-month period (June-November) in 2009. This center, located in Berck-sur-Mer, France, is organized in five wards corresponding to five different clinical specialties (geriatrics, neurology, nutrition, orthopedics and post–operative cares). Such a facility was chose for several reasons. These centers are viewed as a potential reservoir of antibiotic-resistant bacteria for acute-care centers, with the potential for amplification and dissemination into the community. Second, the average stay of 3 months provided a longer follow-up of the patients, thereby improving the study feasibility in terms of wireless recording of interactions among participants.

To enhance the participation of patients and healthcare workers (HCWs), several information meetings were help in April and May 2009. The overall participation rate was 90.1% (788 participants total) of all patients and HCW present in the hospital over the 6–month study period: 452 distinct patients stayed in the hospital, 108 auxiliary nurses, 76 nurse interns, 58 nurses, 28 reeducation therapists (including physiotherapists and ergotherapists), 19 ancillary hospital staff, 7 nurse managers, 7 physicians, and 40 administrative staff. Overall, 8,599 participant-weeks of follow-up were recorded. Each participant was followed for an average of 12.5 weeks (7.6 for patients, 15.2 for HCWs) and up to a maximum of 22 weeks.

## Bacterial follow-up and characterization

Microbiological monitoring of a participating patient included weekly nasal and rectal swabs, as well as swabs of entry wounds or invasive devices (tracheotomy, gastrostomy, *etc*.) for *Staphylococcus aureus* and enterobacteria resistant to third-generation cephalosporins. Screening of participating HCWs included only weekly nasal swabs. All collected swabs (nasal, rectal, and entry wounds combined) were frozen at -80°C and stored in the Microbiological Laboratory of Raymond Poincaré University Hospital (Garches, France). The targeted microorganisms were isolated, subjected to antibiotic-resistance phenotyping and genotyped (to date only *S. aureus*).

## Microbiological methods for *S. aureus* identification and characterization

Nasal swabs collection was performed as described previously with sterile cotton-wool swabs[1]. They were immediately placed in transport medium and sent to the Microbiology Laboratory where they were inoculated into 500 μl of brain-heart infusion medium. Then, 100 μl of this broth were plated onto Chapman agar (Biomérieux®, Marcy l’Etoile, France) and MRSA ID screening agar (Biomérieux®) and incubated for 48 h at 36°C. *S. aureus* was identified by mass spectrometry using the MALDI-TOF-MS spectrometer with flex control software (Autoflex; Bruker Daltonics, Bremen, Germany)

Antimicrobial susceptibility of all isolated *S. aureus* strains was determined on Mueller-Hinton agar plates (BioMerieux®) using the disk diffusion method[2]. The screening procedure had an expected sensitivity of 61.5% and specificity of 98.8%,[3] although higher sensitivity value has been reported (~80%).[4] Twenty antibiotics were tested, including methicillin, which was subsequently used to classify strains as methicillin-resistant (MRSA) or methicillin-sensitive (MSSA). Antibiotic resistance was described as Sensitive-Intermediate-Resistant.

To describe *S. aureus* clonal diversity, all strains isolated from the first positive nasal swab were *spa* typed by Genoscreen (Lille, France) as previously described[5,6]. Briefly, Polymerase Chain Reaction used primers 1113F (TAAAGACGATCCTTCGGTGTGAGC) and 1496R (TTTGCTTTTGCAATGTCATTTACTG) in the following cycle conditions: 12 min at 95°C, 40 cycles: 30 s at 95°C, 30 s at 62°C, 30 s at 72°C, 10 min at 72°C and 15 min at 15°C. DNA sequences were obtained with a 3730xl DNA Analyzer (Applied Biosystems). *Spa* types and minimum spanning tree were determined with Bionumerics v6.5 software (Applied Maths, Saint Martens, Belgium). A broad diversity of *spa* types were isolated, denoting a high variability in *S. aureus* strains (Fig. 1).

## Close proximity interaction recording

We obtained large-scale dynamic networks of interactions among patients and HCWs within the hospital. These interactions were measured by equipping all study participants with a “LogSensor” (WSN430): a wireless device to signal and record the presence of others LogSensors devices in their short radio-frequency range. Our team developed the LogSensor hardware and software. LogSensor is powered by a TI MSP430 micro-controller and communicates through a TI Chipcon CC1100 packet radio interface. The sensor design is classical (see Fig. 2). Interactions between two sensors are recorded by broadcasting periodical beacons (“Hello” packets). When a node *X* received a “Hello” packet from another node *Y,* it logged that at a *Y* to *X* interaction had occurred at local time *t*. The software was implemented as a loop performing simple operations: (i) periodically sending/receiving “Hello” packets; (ii) uploading the data when the memory was almost full. The most restrictive element was battery life. Therefore, sensors were kept in a low-power mode (sleep mode) for most of the time, and only awakened to send and receive information (Fig. 3). Using a sleep period extended the battery longevity but also increased the probability of missing incoming “Hello” packets. Therefore the software allowed for discovery of all nodes that remained at a distance < 1.5 m during a given period, and for those trying to send their beacons at the same time.

## Deployment details

LogSensors were distributed in batches during the first 5 weeks of the investigation (1 week for each ward). All participants within the hospital (medical & administrative staff, patients) were asked to keep their LogSensors with them continuously during their presence in the hospital. For HCWs, the sensor was kept in the overcoat pocket. For patients, it could be in a pocket, worn as a watch or around an ankle and was placed at bedside during the night. For patients unable to move, it was attached to the bed.

Five hundred and eleven LogSensors were distributed during the investigation period and 817 participant files were recorded. Each participant’s file contained data for up to 22 weeks of follow-up. During the study period, recording was discontinued on three occasions for 1 or 2 days to change the sensor batteries. In case of battery failure between changes, the interactions were not recorded for this participant until next scheduled battery change.

## LogSensor design, calibration and management

At the start of the experiment, each LogSensor’s memory was empty and a sensor only knew its unique identifier. Thereafter, sensors distributed to all experiment participants periodically (every 30 s) recorded their surrounding neighborhood. We expected recordings of close interactions, i.e., LogSensors within 1.5-m radius. From experiments made using such sensors, in a clean environment (line of sight), the recordings can vary significantly between two sensors due to hardware-components variations and tolerance. For example, in some experiments using a set of 3 equidistant sensors (60 cm), received signal-strength indication (RSSI) ranged from ~-57 dBm to ~-47 dBm. In the i-Bird experiment, sensors were carried by participants and body-water content was able to efficiently block the 2.4GHz signal, thus allowing the recording of face-to-face interactions only. Because we are interested in close contact, we concentrated only on high-RSSI signals (Fig. 4).

We chose to record the mean RSSI of all consecutive “Hello” packets received from a given sensor. All the data recorded were saved in the sensors’ onboard memory, meaning that no one could get all the data without having physical access to the sensors during the experiment. To sustain a 6-month-long experiment, data were compressed: instead of recording all contacts one by one, consecutive recordings between two sensors were grouped, allowing one packet loss and keeping only the mean RSSI of all consecutive packets.

To ensure experiment parameters, the LogSensors could also be controlled wirelessly by a management PC. This additional feature cannot be used to retrieve the recorded data but to monitor the good functioning of the sensors that crossed paths during the day (battery levels, anomalies). This information was not recorded and only the last “Hello” packet transmission was used to detect faulty sensors. Management PCs were deployed in specific hospital rooms and only required access to the local area network to exchange data with each other.

# References

1. Alvarez AS, Remy L, Allix-Béguec C, Ligier C, Dupont C, Leminor O, et al. Patient nostril microbial flora: individual-dependency and diversity precluding prediction of Staphylococcus aureus acquisition. Clin Microbiol Infect. 2014;20: 70–78. doi:10.1111/1469-0691.12208

2. Leclercq R, Cantón R, Brown DFJ, Giske CG, Heisig P, MacGowan AP, et al. EUCAST expert rules in antimicrobial susceptibility testing. Clin Microbiol Infect. 2013;19: 141–160. doi:10.1111/j.1469-0691.2011.03703.x

3. Wassenberg MWM, Kluytmans JAJW, Bosboom RW, Buiting AGM, van Elzakker EPM, Melchers WJG, et al. Rapid diagnostic testing of methicillin-resistant Staphylococcus aureus carriage at different anatomical sites: costs and benefits of less extensive screening regimens. Clin Microbiol Infect. 2011;17: 1704–1710. doi:10.1111/j.1469-0691.2011.03502.x

4. Struelens MJ, Hawkey PM, French GL, Witte W, Tacconelli E. Laboratory tools and strategies for methicillin-resistant Staphylococcus aureus screening, surveillance and typing: state of the art and unmet needs. Clin Microbiol Infect. 2009;15: 112–119. doi:10.1111/j.1469-0691.2009.02698.x

5. Frénay HME, Bunschoten AE, Schouls LM, Leeuwen WJ van, Vandenbroucke-Grauls CMJE, Verhoef J, et al. Molecular typing of methicillin-resistant Staphylococcus aureus on the basis of protein A gene polymorphism. Eur J Clin Microbiol Infect Dis. 1996;15: 60–64. doi:10.1007/BF01586186

6. Shopsin B, Gomez M, Montgomery SO, Smith DH, Waddington M, Dodge DE, et al. Evaluation of Protein A Gene Polymorphic Region DNA Sequencing for Typing of Staphylococcus aureus Strains. J Clin Microbiol. 1999;37: 3556–3563.

# Figures

**Figure 1 – Numbers of *spa* types isolated from patients as a function of time in the hospital.** The decrease in the last 4 weeks corresponds to the progressive stop in systematic swabbing due to patient discharge without replacement.

**Figure 2** **– The LogSensor design for the i-Bird experiment.** We used the eZ430-RF2500 board provided by TI (red), that incorporates a highly integrated ultra-low-power MSP430 MCU with 16-MHz performance (MSP430F2274), a 2.4-GHz wireless transceiver (CC2500) and a chip antenna. We added on our own board a 64-Megabytes serial flash memory and a real time clock with a built-in 32,768 kHz oscillator. The sensor is powered by a 3.6 V primary lithium-thionyl chloride (Li-SOCl2) high-energy density 1⁄2 AA-size battery.


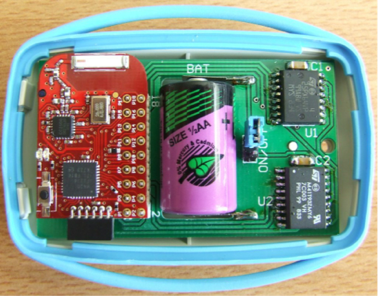


**Figure 3 – « Hello » protocol with sleep period.** Each sensor can be in one of the following three states: listening, talking or sleeping. These three states are present inside a timeframe *F*, of duration *w* + *s*. During *F*, a sensor randomly picks an instant *t_w_*, such that t_w_ ∈ [0 ; *s*]. Then, the sensor randomly selects a second instant *t_i_*, such that *t_i_* ∈ [*t_w_* ; *t_w_* + *w* − *d*]. The sensor is in talking state during [*t_i_* ; *t_i_* + *d*], in listening state during [*t_w_* ; *t_w_* + *w*] \ [*t_i_* ; *t_i_* + *d*] and in sleeping state the rest of the timeframe *F*. The “Hello” message is transmitted at *t_i_* with duration *d*. For each timeframe *F*, the node transmits only one message of duration *d*, listens to its surroundings during *w* − *d* and sleeps during *s*. The medium access probability is therefore: *P* = *d* / (*w* + *s*).


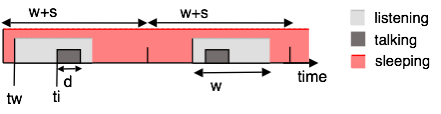


**Figure 4 – Filtering based on the distribution of mean RSSI signals as a function of contact duration.** Since the wireless channel is very versatile, we will only consider packets having a strong RSSI (> -40 dBm). All other packets (gray line near -80 dBm) will be considered as noise.


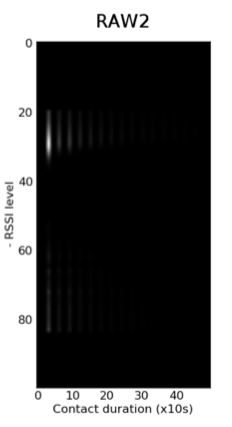

Supplement: S1 Text — (DOCX) [file pcbi.1004170.s001.docx]
