# Supplementary material for: Detailed Contact Data and the Dissemination of Staphylococcus aureus in Hospitals
Source: PLoS Comput Biol. 2015 Mar 19;11(3):e1004170. doi: 10.1371/journal.pcbi.1004170 (PMC4366219; doi:10.1371/journal.pcbi.1004170)
Supplement: S2 Text — (DOCX) [file pcbi.1004170.s002.docx]

# S2 Text: Description of the dynamic CPI network

## Supplementary Methods: building the dynamic CPI network

Individual sensor records were extracted and provided raw contact data. The raw data was cleaned by dropping low-intensity signals, removing parasite records using HCWs work schedules and symmetrizing records to correct for occasional recording glitches. Finally, we transformed the consecutive records with the same sensor in start/duration data, and cumulated the time of interaction between pairs by day. This led to the definition of a dynamic network of 120 successive daily CPI networks, where nodes were the participants and an edge existed when a CPI lasting ≥ 30 s existed during a day. For each patient admitted during the study period, we recorded the number of days to the first direct CPI with others in the hospital. The cumulative number of those neighbors in the CPI network was computed as a function of time since admission, as well as that of 2-hop neighborhoods (i.e. neighbors of neighbors) and 3–hop neighborhoods (see Fig. 5).

The percentage of between-wards CPIs was compared to that expected if the same CPIs had been random between all participants using a Chi-square test.

The daily numbers of patients’ CPIs during the first month after admission was studied by using a random-effects linear model, including a random effect for date and individual according to the equation *Y_it_* = *μ* + *Id_i_* + *D_t_* + *e_it_*, where *Y_it_* was the number of CPIs for individual *i* on day *t*, *μ* the average number of CPIs, *Id_i_* the individual-specific random effect, *D_t_* the day and *e_it_* an error term. HCWs’ CPIs were investigated using a similar random-effects linear model where *D_t_* corresponded to the day of the week. The existence of a between-individual variability was tested by comparing the between-subjects variance to 0 with a restricted likelihood ratio test.[1]

## Dynamics of the CPI network

The dynamic CPI network among individuals in the hospital was dense: a CPI occurred in 19.3% of the 120,735 potential daily interactions between pairs of individuals. Most contacts occurred within wards (81.7% CPIs within wards vs. 29.5% expected by chance, *P* < 0.0001; Fig. 1 in main text), yet most participants also had CPIs in another ward during their time in-hospital (97%, 481 out of 495 affected to a ward). CPIs outside the ward were highly heterogeneous: 80% of all between-wards CPIs were attributable to only 40% of all participants. The rate of outside-the-ward CPIs was higher for patients than HCWs (2.8 vs. 2.1/person per day, *P* = 0.016).

The degree distribution in the daily CPI networks was right-skewed, indicating that while most individuals had CPIs with a limited number of neighbors on each day, a few had a large number of CPIs (Fig. 6). On a given day, the shortest path between any two individuals had 5 ± 3 (mean ± SD) intermediaries, much fewer than the number of individuals in the network. Patients had CPIs with 12 (± 6.2) distinct persons each day, half of them with other patients (n = 6 ± 4.5) and the others with HCWs (n = 6 ± 2.5); whereas HCWs had CPIs with 15 (± 7.2) persons, among whom 9 (± 5.7) were patients and 6 (± 3.1) other HCWs. The corresponding median numbers of CPIs were 9 (range: [1-98]) for patients and 12 (range [1-84]) for HCWs. While the numbers of CPIs were within the same range for patients and HCWs, the daily-cumulative durations of the CPIs were much longer for patients than HCWs, respectively: 12.2 (± 11.3) h vs. 3.7 (± 2.4) h (Fig. 2 in main text). Defining a participant’s *n*-hop neighborhood as all individuals within *n* steps from him revealed that a majority of people in the hospital was within the 3-hop neighborhood of any participant, another illustration of the small-world characteristics of the CPI network (Fig. 5).

Patients with the highest CPI frequencies tended to have more CPIs than other patients every day: the between-patient variability accounted for 36% of the total CPI number variance, more than the day-to-day variation for the same patient (25% of the variance). Likewise, the cumulative CPI durations varied widely among participants, with coefficients of variation (standard deviation/mean) of 92% for patients and 66% for HCWs. The number of distinct persons with whom CPIs occurred increased quickly with the time since admission, reaching 50% of its final expanse after only 5 days (Fig. 5). However, these values varied markedly among patients, both regarding the final number of neighbors (3 to 246) and the time to acquire them (0 to 108 days). Each patient’s extended network, including 2-hop (neighbors of neighbors) and 3-hop neighborhoods, grew accordingly.

# References

1. Scheipl F, Greven S, Küchenhoff H. Size and power of tests for a zero random effect variance or polynomial regression in additive and linear mixed models. Comput Stat Data Anal. 2008;52: 3283–3299. doi:10.1016/j.csda.2007.10.022

# Figures

**Figure 5 – Change of patients’ neighborhood during the first month after inclusion. (Left)** *n*-hop neighborhood includes all individuals whose shortest CPI path from the *ego* (yellow) is smaller or equal to *n*. Lines show the 1-hop neighborhood (solid), 2-hop (dashed) and 3-hop (dotted). Patients are shown as triangles and HCWs as diamonds. **(Right)** Mean size of patients’ neighborhoods during the first month of hospitalization (1-hop: solid, 2-hop: dashed, 3-hop: dotted, maximum: bold). The maximum neighborhood includes all individuals that could have been contacted on each day. The gray area limits the 2.5% to 97.5% percentile of the 1-hop neighborhood size distribution.

**Figure 6 – Degree distribution in patients and HCWs.** Patients are shown as blue triangles and HCWs as red diamonds.
